# Modeling of the Effective Permittivity of Open-Cell Ceramic Foams Inspired by Platonic Solids

**DOI:** 10.3390/ma14237446

**Published:** 2021-12-04

**Authors:** Jesus Nain Camacho Hernandez, Guido Link, Markus Schubert, Uwe Hampel

**Affiliations:** 1 Institute of Fluid Dynamics, Helmholtz-Zentrum Dresden-Rossendorf, 01328 Dresden, Germany; j.hernandez@hzdr.de (J.N.C.H.); u.hampel@hzdr.de (U.H.); 2 Institute for Pulsed Power and Microwave Technology IHM, Karlsruhe Institute of Technology, 76344 Eggenstein Leopoldshafen, Germany; 3 Chair of Imaging Techniques in Energy and Process Engineering, Technische Universität Dresden, 01062 Dresden, Germany

**Keywords:** open-cell ceramic foams, microwave heating, effective permittivity, Platonic structures, complex permittivity

## Abstract

Open-cell solid foams are rigid skeletons that are permeable to fluids, and they are used as direct heaters or thermal dissipaters in many industrial applications. Using susceptors, such as dielectric materials, for the skeleton and exposing them to microwaves is an efficient way of heating them. The heating performance depends on the permittivity of the skeleton. However, generating a rigorous description of the effective permittivity is challenging and requires an appropriate consideration of the complex skeletal foam morphology. In this study, we propose that Platonic solids act as building elements of the open-cell skeletal structures, which explains their effective permittivity. The new, simplistic geometrical relation thus derived is used along with electromagnetic wave propagation calculations of models that represent real foams to obtain a geometrical, parameter-free relation, which is based only on foam porosity and the material’s permittivity. The derived relation facilitates an efficient and reliable estimation of the effective permittivity of open-cell foams over a large range of porosity.

## 1. Introduction

Open-cell foams are use in thermal engineering as heaters and thermal dissipaters [1,2,3]. When they are used as heaters, energy has to be used to heat them. A promising approach for heating such foams is by using microwave radiation. This can be achieved with skeletons made of susceptor materials, such as dielectric ceramics. In this context, their permittivity is important, since it is a key aspect of the polarization and electromagnetic energy dissipation of the material that is exposed to an incident electromagnetic wave (e.g., microwaves). The dissipation of electromagnetic energy, which is responsible for the material heating, is caused by the imaginary part of the permittivity, known as the dielectric loss. The complex permittivity is defined as:

(1) $$\varepsilon =\varepsilon^{\prime }-j\varepsilon^{\prime \prime }\;\mathrm{with}\;\varepsilon^{\prime \prime }=\varepsilon^{\prime }\tan\delta ,$$

where $\varepsilon^{\prime }$ is the dielectric constant, $\varepsilon^{\prime \prime }$ is the dielectric loss, and tanδ is the loss tangent. 

**Figure 1:**
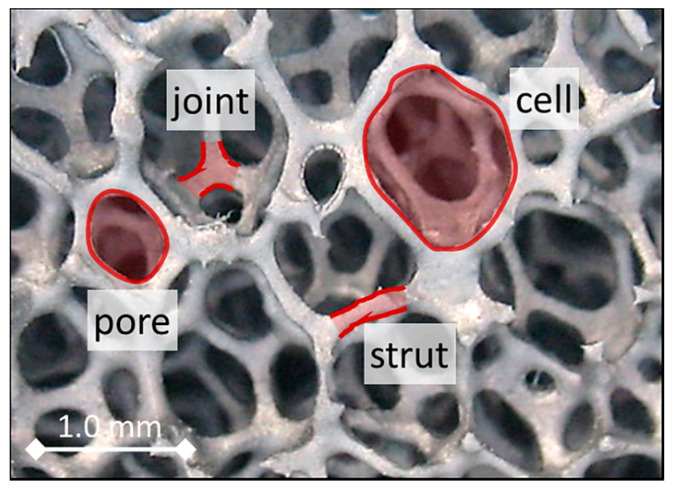
SiSiC open-cell foam with characteristic structural elements (reconstructed µCT image).

Open-cell foams are heterogeneous mixtures, composed of a skeleton that is confined in a continuous medium (e.g., air, water, or even vacuum). The skeleton of open-cell solid foams is a continuous grid of struts, which are interconnected via vertices (as shown in Figure 1). Their morphology depends on the number, shape and spatial arrangement of these struts and junctions, which result from the manufacturing technique that is applied. As the structural elements (e.g., pores, cells, struts and joints; see Figure 1) of foams are much smaller than the wavelength of the incident microwave radiation, the foams tend to behave similarly to a homogeneous media. Consequently, the mixture can be considered, from a macroscopic perspective, to be an effective medium. This consideration is known as the effective medium approximation (EMA). A mixture following the EMA is characterized via its effective permittivity. This effective permittivity $\varepsilon_{\mathrm{eff}}$ depends on the porosity P (ratio of the volume of voids to the total volume of the foam), the permittivities of the solid structure $\varepsilon_{c}$ (i.e., ceramic skeleton) and the surrounding continuous medium (i.e., fluid) $\varepsilon_{f}$, as well as on the morphology of the skeletons. However, given the complex morphology of skeletons (see Figure 1), it is hardly possible to fully describe the skeletons with simple geometrical models. As a result, exact equations for calculating the effective permittivity of porous materials are currently not available.

Instead, the following two approaches are used the most to estimate the $\varepsilon_{\mathrm{eff}}$ of complex mixtures:

1. Relations, such as the EMA relations [4,5] or probability distribution relations of the micromechanical bounds (e.g., Wiener bounds) [4], have been derived. Phenomenologically, these relations consider inclusions to be spherical (or quasi-spherical, e.g., ellipsoids) particles that are embedded in a continuous medium. Reliable predictions can only be obtained if the real microstructure resembles features of the one from which the relation had been derived.
2. Numerical approaches consider the real 3D structure of the skeleton, which is, for example, reconstructed from tomographic scans. The real foam structures are subsequently recreated in a 3D simulation environment [6] to help perform numerical electromagnetic calculations to obtain their scattering parameters [7] and the corresponding $\varepsilon_{\mathrm{eff}}$. Here, the accuracy depends on the spatial resolution of the scanned 3D volume and the refinement of the simulation mesh. The superiority of this method for estimating the $\varepsilon_{\mathrm{eff}}$ comes at the expense of imaging and simulation costs.

In this work, we propose a modified approach to determine the effective permittivity of real open-cell solid foams using simplified structures for electromagnetic wave propagation simulations. For that, we model the skeleton of the foam based on a multitude of Platonic solids, which enables an approximation of the complex foam morphology. A new relation for the effective permittivity of open-cell foams is derived and compared with other relations outlined in the literature and experimental data.

## 2. Materials and Methods

### 2.1. Sintered Open-Cell Ceramic Foams 

In this work, two samples of open-cell ceramic foams with pore densities of 20 and 30 ppi (pores per inch), both made of silicon-infiltrated silicon carbide (SiSiC), were used (manufactured by IKTS Fraunhofer, Dresden, Germany, via the replication sintering technique). Note that the pore density is a measure of the interface density, which corresponds to the inverse of the chord length [8]. It is commonly given in form of a ppi by value manufacturers. The skeletons of these samples were replicated numerically as explained below.

**Image acquisition and processing.** Slices of the foam cross-sections were obtained via X-ray microcomputed tomography (IKTS Fraunhofer, Dresden, Germany) (µCT). The reconstructed 2D cross-sectional images were compiled into stacks (size: 40 mm × 40 mm × 25 mm) with a voxel size of 56 μm × 56 μm × 56 μm. These 3D stacks were then post-processed to correct undesirable defects and aberrations as described elsewhere [8]. 

**Figure 2:**
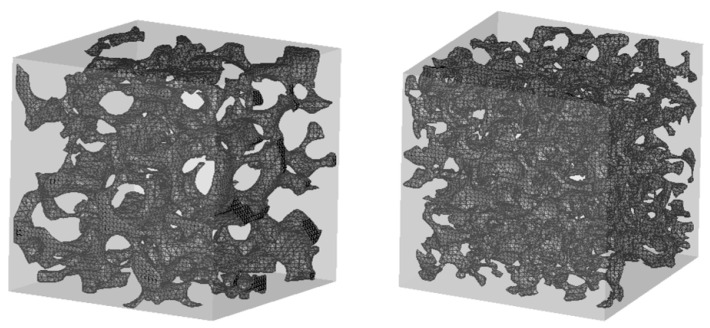
Illustration of the mean representative cubic volume element (MRCV) of the sintered foams (**left**: 20 ppi, **right**: 30 ppi).

**Construction of the model.** To construct representative foam models for the numerical simulations, the size of the 3D stacks was reduced to avoid a high computational burden while preserving the porosity. The stacks were cut to mean representative cubic volume elements (MRCV) with an edge length $L_{\mathrm{MRCV}}$ = 10 mm, at which the porosity still agrees with the original sample porosity. The skeleton morphology was then reconstructed as a 3D mesh by applying the marching cube algorithm (a computer graphics algorithm that calculates triangle vertices from volumetric data by using linear interpolation to render isosurfaces and generate polygonal meshes [9]) to the stacks, followed by post-processing steps, such as mesh smoothing and simplification [10]. Eventually, the reconstructed models of the “sintered foams” (Figure 2) attained porosities of 89.3% (20 ppi) and 89.8% (30 ppi), which agree well with those reported by the manufacturer (88% and 89%, respectively).

Additionally, 3D dilation and erosion filters [11,12] were applied to the original image stacks that were obtained by µCT for adjusting the porosities, followed by the reconstruction process described above. Resulting mean cell diameters $d_{\mathrm{cell}}$ and mean strut diameters $d_{\mathrm{st}}$ that were determined using the 3D thickness method [13] are summarized in Table 1.

### 2.2. Open-Cell Foams Modeled with Platonic Skeletons

**Figure 3:**
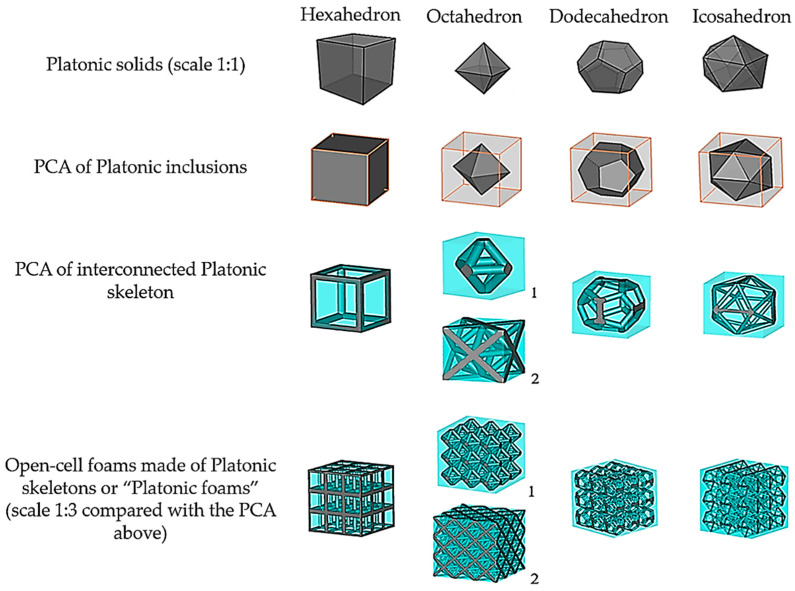
Geometrical structures of open-cell foams built from Platonic skeletons. (1: Octahedron_1_, 2: Octahedron_2_).

Platonic solids are regular polyhedrons with identical faces and equal vertex angles. The polyhedrons that were used in this work were hexahedrons, octahedrons, icosahedrons and dodecahedrons [14]. An important feature of these solids is that they can be inscribed in a cube enclosure, whose outer faces can be considered to be periodic boundaries (see Figure 3). This guarantees that these cubes, when juxtaposed, repeat the Platonic solids with the same spatial arrangement. In the case of octahedrons, two arrangements satisfy their periodicity: octahedron_1_ (the struts cross at the center of the cube faces) and octahedron_2_ (the struts cross at the center and corners of the cube faces). Throughout this article, these arrangements of Platonic solids will be referred to as the periodic cubic enclosure arrangement (PCA) of Platonic inclusions.

**Figure 4:**
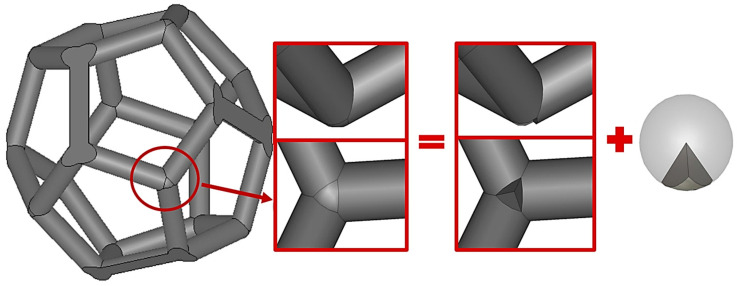
Example of vertex filling for the dodecahedron structure.

**Figure 5:**
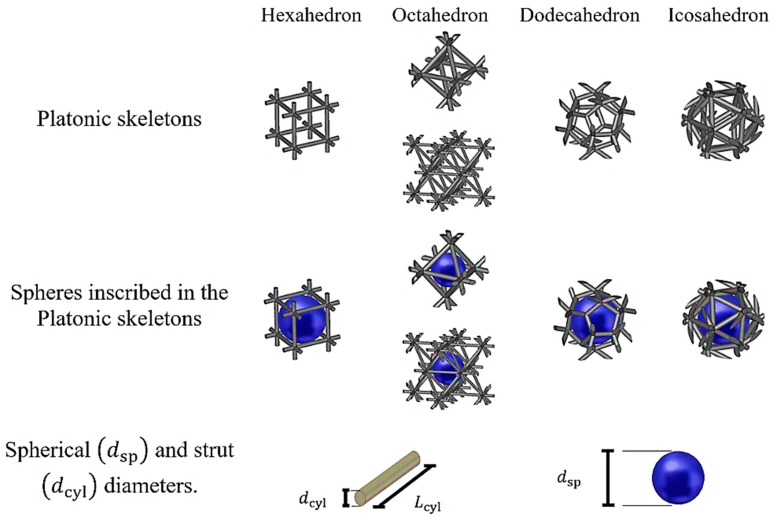
Visual representation of the Platonic foams with the diameters of the strut and inscribed sphere.

The cylinders at the edges of the Platonic solids form an interconnected network that is equivalent to the skeleton of open-cell foams with their struts and joints. However, such a simplification adds a geometrical defect (gap) at the vertices where the cylinders come across each other. These gaps can be easily filled using Boolean operations with a spherical triangle, as shown in Figure 4. In this way, a PCA of interconnected Platonic networks is obtained, which—upon replication in any direction (*x*, *y* or *z*)—forms the open-cell foams of Platonic skeletons that are referred to as “Platonic foams” (see Figure 3, bottom row). Their cylinders represent—by analogy—the struts of sintered foams. A sphere inscribed in the Platonic network—by analogy—is the equivalent of the cell of sintered foams. Moreover, since the geometries of these foams are regular, both their cylinders and spheres have constant diameters, $d_{\mathrm{cyl}}$ and $d_{\mathrm{sp}}$, respectively, as shown in Figure 5. 

Since Platonic foams are composed of equal geometric elements, the volume of the Platonic skeleton $V_{P}$ can be calculated using the diameter $d_{\mathrm{cyl}}$ and length $L_{\mathrm{cyl}}$ of the cylinders. As Table A1 in the Appendix A shows, $L_{\mathrm{cyl}}$ is related to the side length of the PCA $L_{\mathrm{PCA}}$. The porosity (*P* = 1 − skeleton volume fraction) of Platonic foams can be expressed as a function of their structural geometric elements (see Table 2 and Appendix A.1 and Table A1 in the Appendix A). It should be noted that the Platonic foams have an existence limit, since the struts would overlap if $d_{\mathrm{cyl}}$ exceeds a certain size, causing the open-cell faces to close, meaning that the Platonic geometry is no longer preserved. 

### 2.3. Calculation of the Effective Permittivities

**Figure 6:**
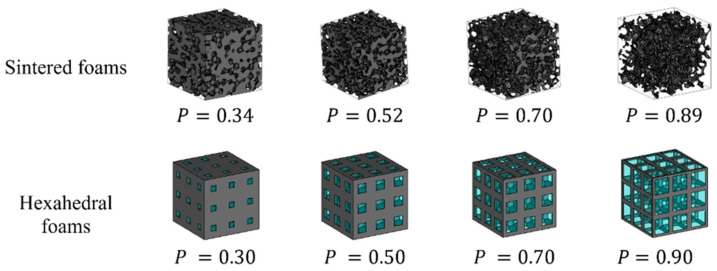
Visual representation of sintered (30 ppi) and Platonic (hexahedral) foams for different porosities.

Sintered and Platonic foam models with different porosities were generated (see Figure 6) to analyze the influence of the morphology on the $\varepsilon_{\mathrm{eff}}$. For the Platonic foams, the diameter of the struts was varied to adjust their porosity so that it was similar to the values of the sintered foams, as shown in Table 1. All of the Platonic foam models were adjusted to a total length L of 10 mm to match $L_{\mathrm{MRCV}}$ of the sintered foams.

These models were then imported into CST Microwave Studio Suite (Version 2018, Dassault Systemes, Velizy-Villacoublay, France) to perform electromagnetic wave propagation simulations with the transient domain solver. This commercial software uses the finite-difference time-domain method to solve the integral formulation of the Maxwell equations. A frequency of 2.45 GHz was chosen for the pulse excitation signal of the microwaves.

**Figure 7:**
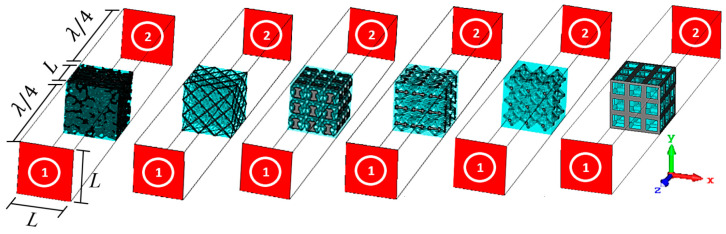
Setup of the numerical simulations to calculate the dispersion parameters (from left to right: sintered, icosahedral, dodecahedral, octahedral_2_, octahedral_1_ and hexahedral foams; red faces represent the ports).

The simulation setup is shown in Figure 7. This approach is identical to the one reported elsewhere [7,10]. The boundary conditions are: normal electric ($E_{\tan\mathrm{gential}}=0$) and magnetic ($H_{\tan\mathrm{gential}}=0$) walls for the *x-* and *y*-axes, respectively; input (1) and output (2) are open boundaries with added space and non-reflective waveguide ports and a distance to the models equal to λ/4 for the *z*-axis.

The loss tangent and dielectric constant of the skeleton were assigned as:

1. $\tan\delta_{c}=0.23$ with $\varepsilon_{c}^{\prime }=10$;
2. $\tan\delta_{c}=0.46$ with $\varepsilon_{c}^{\prime }=20$;
3. $\tan\delta_{c}=0.91$ with $\varepsilon_{c}^{\prime }=40$;

which are doubles of each other. The parameters of the continuous medium were assigned as:

- $\tan\delta_{f}=0$ with $\varepsilon_{f}^{\prime }=1$ (corresponding to the air permittivity);
- $\tan\delta_{f}=0.14$ with $\varepsilon_{f}^{\prime }=2.6$ (as an alternative to air).

The higher $\varepsilon_{f}$ was not chosen to match a particular fluid but rather to evaluate its effect on the effective permittivity.

By moving the reference planes of the ports to the faces of the models, the reflected *S*_11_ and transmitted *S*_21_ scattering parameters were acquired. These scattering parameters are used in the retrieval method [15] to calculate the effective permittivity of each model. This is achieved by calculating the impedance z and the refractive index n as:

(2) $$z=\pm \sqrt{\frac{{\left(1+S_{11}\right)}^{2}-S_{21}^{2}}{{\left(1-S_{11}\right)}^{2}-S_{21}^{2}}},$$

(3) n=1k0L[Im{ln(S211−(z−1)S11(z+1))}+2mπ−i·Re{ln(S211−(z−1)S11(z+1))}],

where L is the model length between the reference ports, $k_{0}$ is the wavenumber in free space, and *m* is the fundamental branch of the sinusoidal function periodicity (m=0 for L<λ/4, otherwise m=±1, ±2, …, ±∞). Impedance and refractive index are then used to calculate the effective permittivity $\varepsilon_{\mathrm{eff}}=n/z$ and the effective permeability $\mu_{\mathrm{eff}}=nz$. Since the models have no magnetic properties, $\mu_{\mathrm{eff}}$ was only used to validate whether the impedance and refractive index yield the permeability of a non-magnetic sample, i.e., $\mu_{\mathrm{eff}}=1.0-j0.0\pm \left(0.10-j0.02\right)$.

For the numerical simulations, hexagonal meshes were used. The mesh size for the Platonic foams was defined based on a sensitivity study that was performed for the hexahedral foam. The objective was to achieve a mesh size for which the $\varepsilon_{\mathrm{eff}}$ does not change more than 1% and which produces a tolerable computational burden. Cell sizes < 133 µm (with at least $3.4\times 10^{6}$ cells) were assigned for all Platonic foam meshes. Meshes with at least $1\times 10^{7}$ cells were considered for the sintered foams based on the results of a previous study [16]. Note that the PCA is a unit periodic structure, and simulations carried out on one PCA model with periodic boundaries produce results that are consistent (i.e., do not differ by more than 0.5%) to the results that were obtained from the models that are presented in Figure 7.

### 2.4. Expressions for Discussion of Results

The foams that are composed of mixtures of both skeleton and continuous medium materials are analyzed in the discussion section using the real and imaginary susceptibility ratios of the permittivity, expressed as:

(4) SRRe=Re(εeff)−Re(εf)Re(εc)−Re(εf),

(5) SRIm=Im(εeff)−Im(εf)Im(εc)−Im(εf),

and the complex dielectric contrast:

(6) $$\frac{\varepsilon_{c}}{\varepsilon_{f}}=\frac{\varepsilon_{c}^{\prime }\varepsilon_{f}^{\prime }+\varepsilon_{c}^{\prime \prime }\varepsilon_{f}^{\prime \prime }}{\varepsilon_{f}^{\prime }{}^{2}+\varepsilon_{f}^{\prime \prime }{}^{2}}-j\frac{\varepsilon_{c}^{\prime \prime }\varepsilon_{f}^{\prime }-\varepsilon_{c}^{\prime }\varepsilon_{f}^{\prime \prime }}{\varepsilon_{f}^{\prime }{}^{2}+\varepsilon_{f}^{\prime \prime }{}^{2}}.$$

Accordingly, the complex dielectric contrasts of the foam mixtures are:

1. Skeleton: $\tan\delta_{c}=0.23$ with $\varepsilon_{c}^{\prime }=10$, continuous medium: $\tan\delta_{f}=0$ with $\varepsilon_{f}^{\prime }=1,$
  $\varepsilon_{c}/\varepsilon_{f}=10-j2.3$.
2. Skeleton: $\tan\delta_{c}=0.46$ with $\varepsilon_{c}^{\prime }=20$, continuous medium: $\tan\delta_{f}=0$ with $\varepsilon_{f}^{\prime }=1,$
  $\varepsilon_{c}/\varepsilon_{f}=20-j9.2$.
3. Skeleton: $\tan\delta_{c}=0.91$ with $\varepsilon_{c}^{\prime }=40$, continuous medium: $\tan\delta_{f}=0$ with $\varepsilon_{f}^{\prime }=1,$
  $\varepsilon_{c}/\varepsilon_{f}=40-j36.4$.
4. Skeleton: $\tan\delta_{c}=0.46$ with $\varepsilon_{c}^{\prime }=20$, continuous medium: $\tan\delta_{f}=0.14$ with $\varepsilon_{f}^{\prime }=2.6,$
  $\varepsilon_{c}/\varepsilon_{f}=8-j2.4$.

## 3. Results

**Figure 8:**
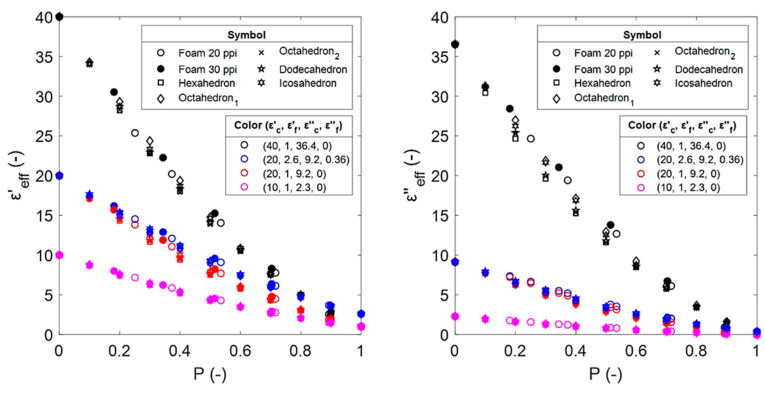
Estimated effective dielectric constants (**left**) and effective dielectric losses (**right**) from numerical calculations for sintered and Platonic foams. The mixtures of the foams from Section 2.2 are identified by their corresponding properties $(\varepsilon_{c}^{\prime }$, $\varepsilon_{f}^{\prime },\;\varepsilon_{c}^{\prime \prime },\varepsilon_{f}^{\prime \prime }$ ).

The results of the numerical calculations for the estimated effective permittivity of the sintered and Platonic foams are shown in Figure 8. Note that due to the different methods that were used to obtain both the sintered and Platonic foams (Section 2.1 and Section 2.2), the models do not have the same porosities.

In general, the difference in $\varepsilon_{\mathrm{eff}}^{\prime }$ and $\varepsilon_{\mathrm{eff}}^{\prime \prime }$ for each foam is observed as a result of their different microstructures. Consequently, the microstructures of the Platonic foams that better represent sintered foams would provide more accurate estimations of the $\varepsilon_{\mathrm{eff}}$. A more detailed discussion of the results is provided in Section 4.

## 4. Discussion

### 4.1. Analysis of the Effective Medium Approximation Applied to Sintered Foams

**Figure 9:**
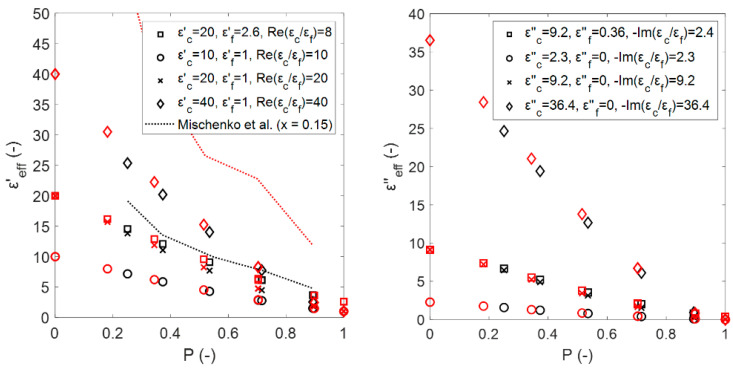
Effective dielectric constants (**left**) and effective dielectric losses (**right**) as a function of porosity, obtained from numerical simulations for the sintered foams with pore densities of 20 ppi (black symbols) and 30 ppi (red symbols). Predicted thresholds (**left**) for the EMA assumption are shown for 20 ppi (black line) and 30 ppi (red line).

As shown in Figure 9, the effective permittivities of the sintered foams (20 and 30 ppi) are practically the same, despite the difference in the size of their struts and cells. Such results provide evidence that the EMA consideration is plausible and that it can be verified through the inclusion size condition. This condition uses an inclusion parameter x expressed as:

(7) $$x=\frac{\pi d_{\mathrm{incl}}}{\lambda },$$

(8) $$\lambda =\frac{\lambda_{0}}{\sqrt{\varepsilon_{\mathrm{eff}}^{\prime }}},$$

where the inclusion diameter $d_{\mathrm{incl}}=d_{\mathrm{cell}}$ (by analogy) and $\lambda_{0}$ is the wavelength in the air ($\lambda_{0}≅12.2$ cm at 2.45 GHz) of the incident radiation. Then, x is compared with a threshold value that is known to meet the standards for EMA behavior. Using the inclusion size parameter (Equations (4) and (5)) and the threshold value x = 0.15, as implied from the results of Mishchenko et al. [17], it is possible to validate the EMA consideration in terms of the $\varepsilon_{\mathrm{eff}}^{\prime }$ as:

(9) $$\varepsilon_{\mathrm{eff}}^{\prime }<<{\left(\frac{0.15\lambda_{0}}{\pi d_{\mathrm{cell}}}\right)}^{2}.$$

The value of x = 0.15 relates to the particle size at which the dispersion matrix (of the refractive index) of the host-inclusion mixture differs from the dispersion matrix that is calculated using the Lorenz–Mie computations that are based on EMA relations [17]. Figure 9 (left) shows the effective permittivity (marked with symbols) that was obtained from the numerical simulations for the sintered foams compared to the predicted thresholds for the EMA assumption (represented as lines) in accordance with the inclusion size condition. The obtained data illustrate that the EMA assumption is valid except for some points of the skeletons with $\tan\delta_{c}=0.91$ ($\varepsilon_{c}^{\prime }=40$) and a 20 ppi pore density. As the pore size increases, the inclusion size parameter approaches the threshold value. For those points that have already exceeded the threshold value (at the given effective permittivity), the scattering matrix of the foam no longer accurately reproduces the scattering matrix of an EMA mixture. Therefore, it is expected that the EMA assumption slightly begins to lose its validity, and therefore the $\varepsilon_{\mathrm{eff}}$ of those samples may deviate slightly from the trend of the others.

**Figure 10:**
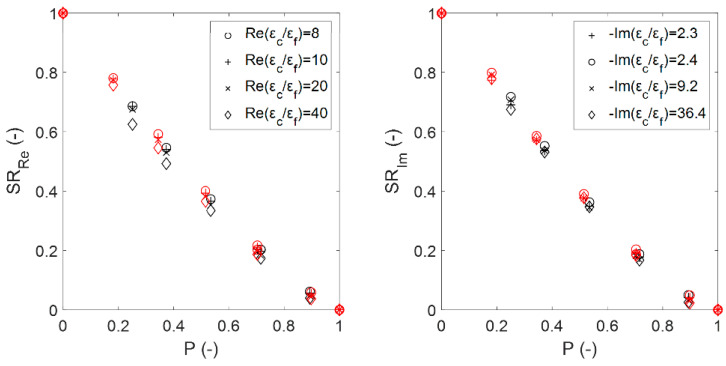
Estimated real (**left**) and imaginary (**right**) susceptibility ratios from numerical simulations for the sintered foams with pore densities of 20 ppi (black symbols) and 30 ppi (red symbols).

Figure 10 shows the effect of the porosity on the susceptibility ratios from the numerical simulations for the sintered foams. These data reveal the contribution of the interactions between the inclusion and the background constituents of the system. For a given porosity (except for $\varepsilon_{\mathrm{eff}}\left(P=0\right)=\varepsilon_{c}$ and $\varepsilon_{\mathrm{eff}}\left(P=1\right)=\varepsilon_{f}$), the effective dielectric constant and complex dielectric contrast do not increase proportionally [5]. When one half-wavelength of the radiation approaches the inclusion size where resonance occurs [18], the propagating fields gradually decrease due to originated evanescent and near fields, which decrease the possible value of $\varepsilon_{\mathrm{eff}}^{\prime }$ as shown for SRRe. The fact that the increase in the dielectric contrast affects SRRe only (and not SRIm) is related to the shortened wavelength that propagates inside the foam, which depends only on the $\varepsilon_{\mathrm{eff}}^{\prime }$ (see also Equation (5)).

### 4.2. Comparison of Effective Permittivity Estimates with Mixing Relations from the Literature

Practically, two approaches are applicable for deriving the relations for the permittivity of the effective media, i.e., the effective model approximation (EMA) [5] and the weighted means of bounds [4] (e.g., the Wiener or Hashin-Shtrikman bounds). Below, the predictive capabilities of relations for open-cell solid foams that are outlined in the literature are analyzed.

The EMA mixing relations are derived from the homogenization theory, where homogeneously distributed inclusions in a quasi-uniform continuous medium are considered. Here, the effective permittivity is estimated based on the porosity and the constituents of the system, using different EMA relations to those given in Table 3 (please note relation abbreviations). 

In turn, in probability distribution relations that are based on the weighted means of bounds, the $\varepsilon_{\mathrm{eff}}$ of homogeneous media with arbitrary microstructures is restricted by micromechanical limits. The Wiener bounds [4]:

(10) $$\varepsilon_{W}^{+}=\left(1-P\right)\varepsilon_{c}+P\varepsilon_{f}\;(\mathrm{upper}\;\mathrm{bound}\;=\;\mathrm{top}\;\mathrm{restriction}),$$

(11) $$\varepsilon_{W}^{-}={\left[\left(1-P\right)/\varepsilon_{c}+P/\varepsilon_{f}\right]}^{-1}\;(\mathrm{lower}\;\mathrm{bound}=\mathrm{bottom}\;\mathrm{restriction})$$

are considered to be appropriate for any mixture, and thus they are applied here to ensure that the effective permittivity lies between the upper and lower bound. Since there is no rigorous model on which these relations are based, weighting parameter Ψ and exponential parameter N are fitted to the experimental data using a non-linear least squares solver [19,20].

**Figure 11:**
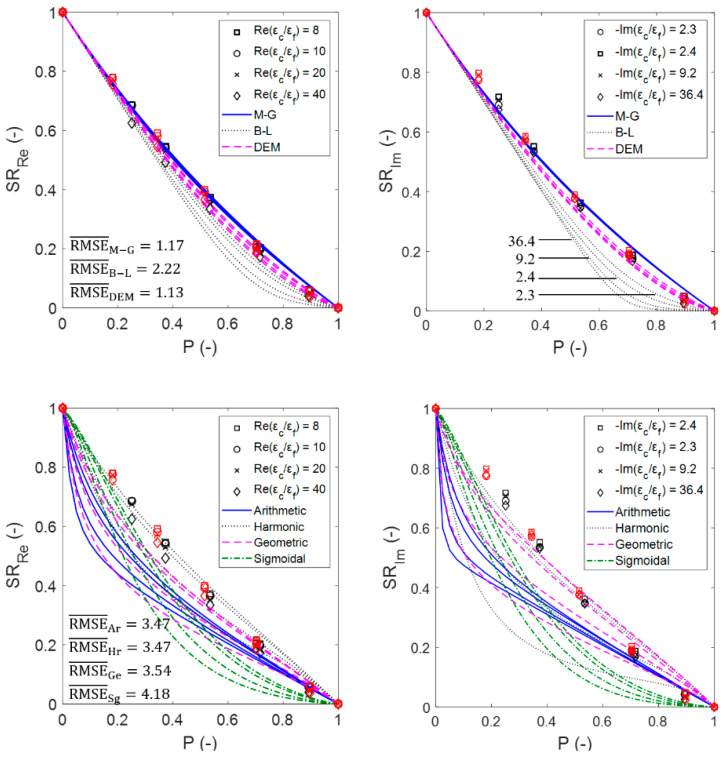
Real (**left**) and imaginary (**right**) susceptibility ratios of the EMA relations (**upper row**) and the probability distribution relations (**lower row**) compared with the calculated ratios from the numerical simulations for sintered foams for 20 ppi (black symbols) and 30 ppi (red symbols). The figure symbol corresponds to the complex dielectric contrasts of the foam mixtures introduced in Section 2.4, while lines illustrate the EMA relations predictions. The curves derived from the relations follow a descending order as $\varepsilon_{c}/\varepsilon_{f}$ increases (as illustrated in the upper right figure for the B-L relation).

Figure 11 shows the susceptibility ratios of the estimated effective permittivity for sintered foams (marked with symbols) compared to those that were obtained using the EMA relations (continuous data shown by lines), as well as the root mean square errors (RMSE) that have been averaged for the dielectric constant and the loss factor as $\bar{\mathrm{RMSE}}=\left({\mathrm{RMSE}}_{\varepsilon^{\prime }}+{\mathrm{RMSE}}_{\varepsilon^{\prime \prime }}\right)/2$. From the obtained data, it can be deduced that the DEM relation best estimates the simulated permittivity of the sintered foams, followed by the M-G relation. This is not unexpected, given that, in general, both of the mixing relations provide very close estimations of the effective permittivity [17]. Unlike the M-G relation, the DEM relation is symmetrical to all of the medium components and therefore treats them all equally. Thus, the DEM relation can produce significantly better estimations in cases where the volume fraction of the inclusions is considerably large, as it is for sintered foams.

### 4.3. Comparison of Effective Permittivity Estimates from Sintered and Platonic Foams

**Figure A1:**
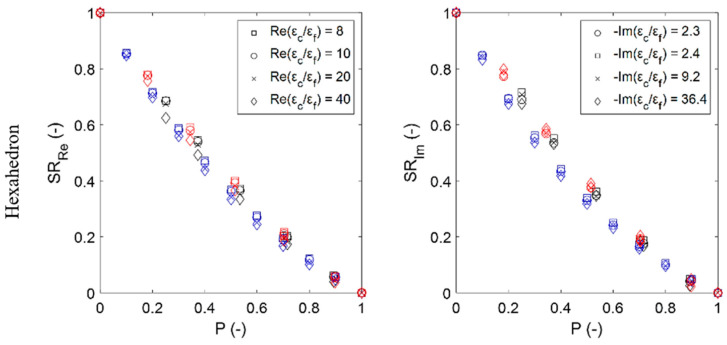

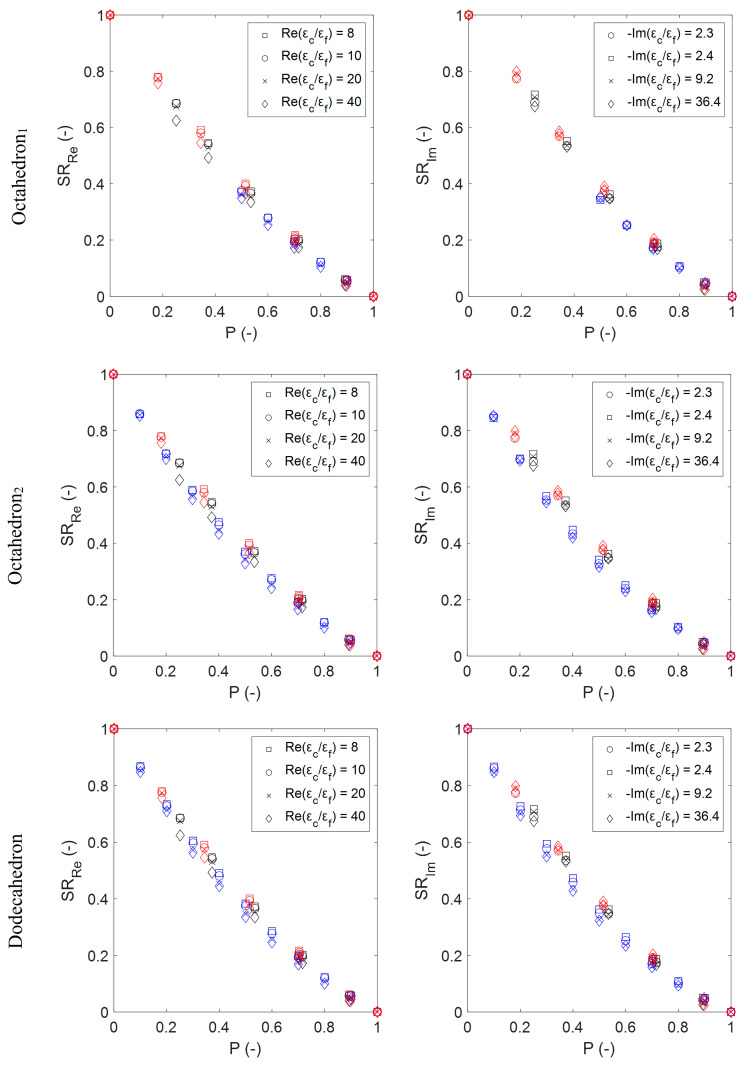

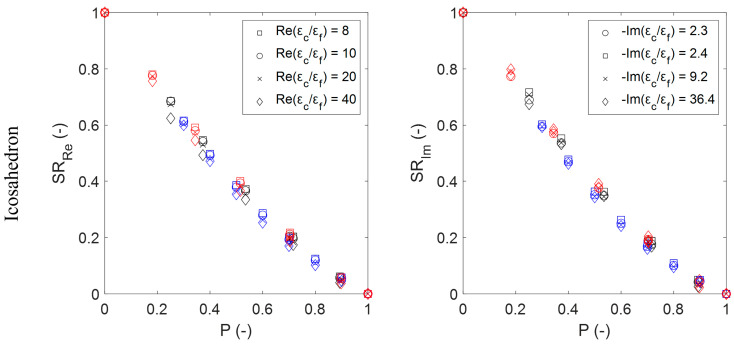
Real (**left**) and imaginary (**right**) susceptibility ratios of the numerical simulations of the Platonic foams (blue symbols) compared with the numerical simulations for the sintered foams at 20 ppi (black symbols) and 30 ppi (red symbols).

The effective permittivities that have been obtained from the electromagnetic wave propagation simulations for the Platonic foams, along with the sintered foams, are shown in Figure A1 of the Appendix A. The effect of the different Platonic solids that were used as building elements of the open-cell skeletal structures is low. However, a general order can be observed for the dielectric loss, i.e., octahedron_1_ > icosahedron > dodecahedron > octahedron_2_ > hexahedron. This coincides with the order in which the Platonic shapes are preserved (from Table 2), i.e., there is a decrease in the size of the faces of the open cells of the Platonic solids by increasing the struts. This suggests that as the open-cell faces become more closed, the resistivity decreases and, in turn, the loss increases. 

### 4.4. Analysis of Proposed Relations for the Estimation of the Permittivity of Platonic and Sintered Foams

**Figure 12:**
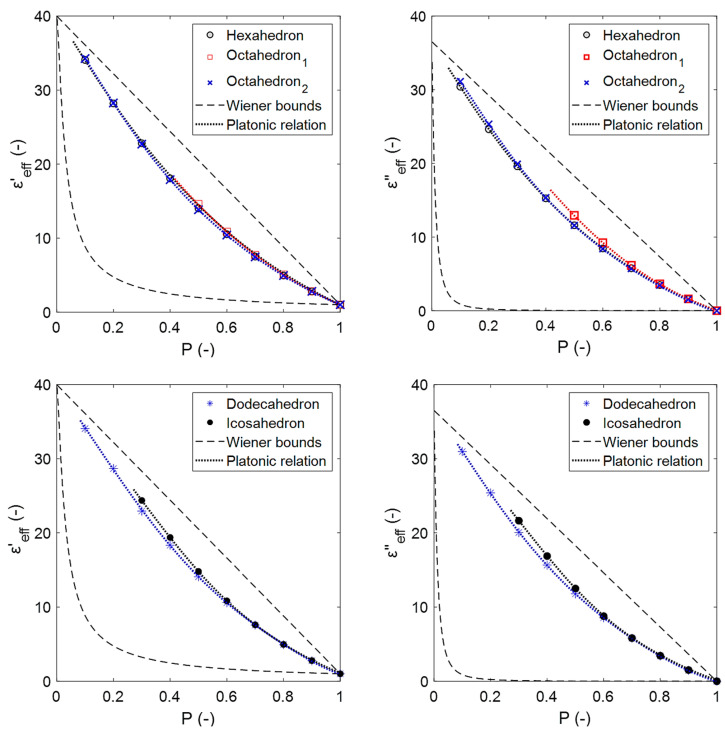
Effective dielectric constants (**left**) and losses (**right**) obtained from simulations for the Platonic foams (colored dot lines were obtained using Equation (23), referred to as a Platonic relation; dashed lines represent the Wiener bounds, $\varepsilon_{c}=40.00-j36.53$, $\varepsilon_{f}=1.00-j0.00$ ).

The mixing relations for the $\varepsilon_{\mathrm{eff}}$ of the Platonic and sintered foams that are based on the analysis of their simulated permittivities were derived in Appendix A.3 and Appendix A.4 of the Appendix A. The proposed relation (Equation (A12) of the Appendix A) for Platonic solids, such as the hexahedron, octahedron, icosahedron and dodecahedron, is:

(12) εeff=−2P(1+P2)(εc−εf)+(εc+εf[[Re(εc/εf)gm′+g0′]+j[Im(εc/εf)gm″+g0″]]),

where $g_{0}^{\prime },\;g_{0}^{\prime \prime },\;g_{m}^{\prime }\;\mathrm{and}\;g_{m}^{\prime \prime }$ are given by a polynomial of degree 6 in terms of their porosity (Equation (A13) from Appendix A), and whose coefficients are summarized in Table A2 of the Appendix A. Figure 12 shows an exemplarily $\varepsilon_{\mathrm{eff}}$ that has been estimated using Equation (12), which represents a fair agreement with the Platonic foam calculations. 

On the other hand, the proposed relation (Equation (A15) of the Appendix A) for open-cell foams (referred to as OCF) is given by a simpler equation as:

(13) $$\varepsilon_{\mathrm{eff}}=\frac{-2P}{\left(1+P^{2}\right)}\left(\varepsilon_{c}-\varepsilon_{f}\right)+\varepsilon_{c}\left(1+P{\left(1-P\right)}^{3/2}\right).$$

**Figure 13:**
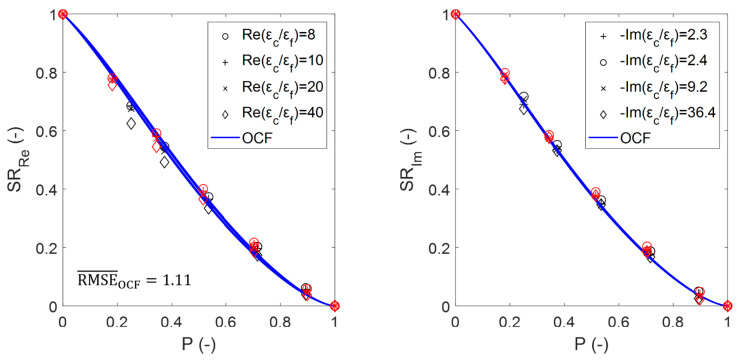
Real (**left**) and imaginary (**right**) susceptibility ratios of the OCF relation compared with the calculated ratios from the numerical simulations for sintered foams for 20 ppi (black symbols) and 30 ppi (red symbols).

Figure 13 shows the susceptibility ratios of the estimated effective permittivity for the sintered foams (marked with symbols) compared to that obtained using the OCF relation (continuous data shown by lines), including the $\bar{\mathrm{RMSE}}$. Figure 13, as well as Figure 11, illustrates the agreement between the data that were obtained from the simulations and relations, which for the OCF relation even exceeds the estimations that were obtained with the DEM relation (${\bar{\mathrm{RMSE}}}_{\mathrm{OCF}}<{\bar{\mathrm{RMSE}}}_{\mathrm{DEM}}$) and thus also for all of the other EMA relations and Platonic foams (see Figure A1). 

**Figure 14:**
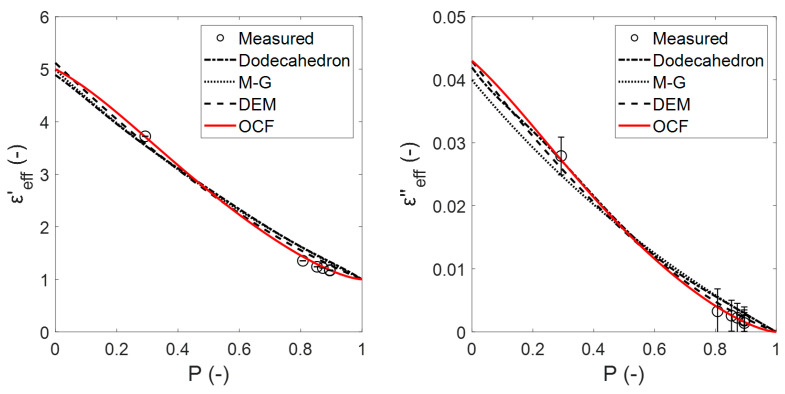
Effective dielectric constants (**left**) and effective dielectric losses (**right**) of cordierite foam samples and the estimates using dodecahedron, OCF, MG, and DEM relations.

**Figure 15:**
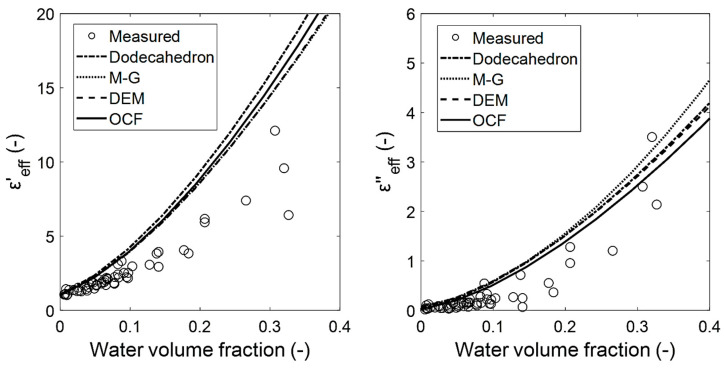
Effective dielectric constants (**left**) and effective dielectric losses (**right**) of polyurethane foam samples and the estimates using dodecahedron, OCF, MG, and DEM relations.

The OCF and Platonic relations were also compared with the experimental data. Unfortunately, the data in the literature are scarce and no records for the variation of the porosity and $\varepsilon_{\mathrm{eff}}$ are available for open-cell foams (keeping their temperature and the frequency of the incident radiation constant). Thus, series of previous $\varepsilon_{\mathrm{eff}}$ measurements (at 2.45 GHz using the cavity perturbation technique) of cordierite (*cord*) samples with 30, 45 and 60 ppi [10] and polyurethane (*poly*) with different moisture (*mois*) content were used for evaluation. The $\varepsilon_{\mathrm{eff}}$ of cordierite and polyurethane, along with the dodecahedron (due to its good fit to the numerical results of the sintered foams) Platonic relation, OCF, M-G and DEM estimations, are shown in Figure 14 and Figure 15, respectively.

In Figure 14, $\varepsilon_{c}$ (unknown bulk permittivity) is obtained from the mixing relations that were fitted to the experimental data using an iterative least-squares estimation. The dodecahedron, M-G and DEM relations show similar estimates but do also have a remarkably different trend compared with the OCF relation. This is due to the different approaches that are used to describe the microstructure of the mixtures (e.g., foams) between relations. All of the relations provide an acceptable estimate 5−j0.04±(0.2−j0.004) of the cordierite bulk permittivity ($\varepsilon_{\mathrm{cord},\max}=6-j0.06$ at P=0 for 20 °C and 1 MHz [21] and $\varepsilon_{\mathrm{cord},\min}=4.77-j0.008$ at P=0 for 20 °C and 8.52 GHz [22]), assuming that the value must be between $\varepsilon_{\mathrm{cord},\min}$ and $\varepsilon_{\mathrm{cord},\max}$ and that this range remains practically constant with the same conditions that were applied in the previous study (100 °C, 2.45 GHz). A better estimation would be obtained by using more points that are equally distributed within the porosity.

Figure 15 shows the $\varepsilon_{\mathrm{eff}}$ of the polyurethane samples (P=0.978±0.007) that were adjusted with different water volume fractions. To estimate the $\varepsilon_{\mathrm{eff}}$, $\varepsilon_{\mathrm{mois}}$ was calculated using the DEM relation with $\varepsilon_{\mathrm{air}}=1.00-j0.00$ and $\varepsilon_{\mathrm{water}}=80.36-j14.57$ [23] (at 2.45 GHz). Next, $\varepsilon_{\mathrm{mois}}$ and $\varepsilon_{\mathrm{poly}}=2.0-j3.2$ (at 2.45 GHz) [24] were used for the mixing relations. For the polyurethane foams, mixing relations were not used (as in Figure 14) to estimate $\varepsilon_{c}$, and thus a different trend of the relations is observed. All of the used relations show a good agreement with each other. The differences to the experimental points may be caused firstly by the fact that water (inclusion) is not homogeneously distributed in the foam voids, and secondly because the most polar polymers, such as polyurethane [25], exhibit a high level of moisture adsorption. Both factors in combination produce a significant variation to an EMA mixture, whereby overestimated values for the effective permittivity are expected. Moreover, at the porosity of P=0.97, $\varepsilon_{\mathrm{eff}}$ is more dependent on $\varepsilon_{\mathrm{mois}}$ than on the foam’s morphology. Finally, it can be concluded that OCF relations provide good estimates that are comparable to those from the EMA relations for $\varepsilon_{\mathrm{eff}}$. 

## 5. Conclusions

In this work, we compared the effective permittivities of fabricated foams (created using the sintering technique) and virtually constructed foams using Platonic skeletons that were calculated using electromagnetic wave propagation calculations. A new relation for evaluating the permittivity of Platonic foams was presented, which can be used for the design of customized foams with desired dielectric properties.

In addition, a new empirical relation was also proposed for estimating the effective permittivity of sintered foams. The relation was obtained through an analysis of the effective permittivity of the sintered foam skeletons. The relation is only based on porosity and bulk material permittivities and does not require any additional empirical or experimental parameters. It has been demonstrated that the new relation for the prediction of the effective permittivity of simulated open-cell foams outperforms any other relation available in the literature. However, more experimental data is still required for validation, especially at a high permittivity contrast where the EMA approach loses its validity.

## Figures and Tables

**Table 1:** Cell and strut diameters of the sintered foams (mean ± standard deviation).

20 ppi	P	0.89	0.72	0.55	0.37	0.25
	$d_{\mathrm{cell}}$ (mm)	2.65 ± 0.51	2.10 ± 0.49	1.85 ± 0.33	1.58 ± 0.28	1.33 ± 0.44
	$d_{\mathrm{st}}$ (mm)	0.64 ± 0.20	0.95 ± 0.21	1.03 ± 0.42	1.41 ± 0.52	1.93 ± 0.23
30 ppi	P	0.89	0.70	0.52	0.34	0.18
	$d_{\mathrm{cell}}$ (mm)	1.68 ± 0.54	1.22 ± 0.20	1.13 ± 0.19	0.92 ± 0.14	0.70 ± 0.17
	$d_{\mathrm{st}}$ (mm)	0.37 ± 0.12	0.53 ± 0.13	0.70 ± 0.30	0.84 ± 0.20	1.22 ± 0.26

**Table 2:** Porosity of the Platonic foams and corresponding limits, where the Platonic geometry is no longer preserved.

Platonic Skeleton	Porosity	Limits for Strut Diameter and Porosity
Hexahedron	$P=1-\left[\frac{3\pi }{4}\frac{d_{\mathrm{cyl}}{}^{2}}{L_{\mathrm{PCA}}{}^{2}}-\sqrt{2}\frac{d_{\mathrm{cyl}}{}^{3}}{L_{\mathrm{PCA}}{}^{3}}\right]$	$d_{\mathrm{cyl}}\leq L_{\mathrm{PCA}}$ P≥0.058
Octahedron_1_	$P=1-\left[\frac{3\pi }{\sqrt{2}}\frac{d_{\mathrm{cyl}}{}^{2}}{L_{\mathrm{PCA}}{}^{2}}-7.73\frac{d_{\mathrm{cyl}}{}^{3}}{L_{\mathrm{PCA}}{}^{3}}\right]$	$d_{\mathrm{cyl}}\leq \frac{L_{\mathrm{PCA}}}{\sqrt{6}}$ P≥0.415
Octahedron_2_	$P=1-\left[\frac{6\pi }{\sqrt{2}}\frac{d_{\mathrm{cyl}}{}^{2}}{L_{\mathrm{PCA}}{}^{2}}-19.31\frac{d_{\mathrm{cyl}}{}^{3}}{L_{\mathrm{PCA}}{}^{3}}\right]$	$d_{\mathrm{cyl}}\leq \frac{L_{\mathrm{PCA}}}{\sqrt{6}}$ P≥0.093
Dodecahedron	$P=1-\left[\frac{27\pi }{2\left(3+\sqrt{5}\right)}\frac{d_{\mathrm{cyl}}{}^{2}}{L_{\mathrm{PCA}}{}^{2}}-9.07\frac{d_{\mathrm{cyl}}{}^{3}}{L_{\mathrm{PCA}}{}^{3}}\right]$	$d_{\mathrm{cyl}}\leq \frac{2L_{\mathrm{PCA}}}{\left(3+\sqrt{5}\right)}\sqrt{1+\frac{2}{\sqrt{5}}}$ P≥0.078
Icosahedron	$P=1-\left[\frac{27\pi }{2\left(1+\sqrt{5}\right)}\frac{d_{\mathrm{cyl}}{}^{2}}{L_{\mathrm{PCA}}{}^{2}}-20.60\frac{d_{\mathrm{cyl}}{}^{3}}{L_{\mathrm{PCA}}{}^{3}}\right]$	$d_{\mathrm{cyl}}\leq \frac{2L_{\mathrm{PCA}}}{\left(\sqrt{3}+\sqrt{15}\right)}$ P≥0.267

**Table 3:** EMA and probability distribution relations.

EMA Relations	Expression
Maxwell Garnett (M-G) (Maxwell-type relation, non-symmetric) [5]	$\frac{\varepsilon_{\mathrm{eff}}-\varepsilon_{c}}{\varepsilon_{\mathrm{eff}}+2\varepsilon_{c}}=\left(P\right)\frac{\varepsilon_{f}-\varepsilon_{c}}{\varepsilon_{f}+2\varepsilon_{c}}$
Bruggeman–Landauer (B-L) (self-consistent relation, symmetric) [5]	$\left(1-P\right)\frac{\varepsilon_{c}-\varepsilon_{\mathrm{eff}}}{\varepsilon_{c}+2\varepsilon_{\mathrm{eff}}}=-\left(P\right)\frac{\varepsilon_{f}-\varepsilon_{\mathrm{eff}}}{\varepsilon_{f}+2\varepsilon_{\mathrm{eff}}}$
Differential effective medium (DEM) (Bruggeman relation, non-symmetric) [5]	$\frac{\varepsilon_{f}-\varepsilon_{\mathrm{eff}}}{\varepsilon_{f}-\varepsilon_{c}}{\left(\frac{\varepsilon_{c}}{\varepsilon_{\mathrm{eff}}}\right)}^{1/3}=1-P$
Probability distribution relations	
Weighted arithmetic (Ar) mean of upper and lower Wiener bounds [4]	$\varepsilon_{\mathrm{eff}}=\left(1-\Psi_{\mathrm{arithm}}\right)\varepsilon_{W}^{+}+\Psi_{\mathrm{arithm}}\varepsilon_{W}^{-}$
Weighted harmonic (Hr) mean of upper and lower Wiener bounds [4]	$\varepsilon_{\mathrm{eff}}={\left[\frac{\left(1-\Psi_{\mathrm{harm}}\right)}{\varepsilon_{W}^{+}}+\frac{\Psi_{\mathrm{harm}}}{\varepsilon_{W}^{-}}\right]}^{-1}$
Weighted geometric (Ge) mean of upper and lower Wiener bounds [4]	$\varepsilon_{\mathrm{eff}}=\exp\left[\left(1-\Psi_{\mathrm{geom}}\right)\ln\left(\varepsilon_{W}^{+}\right)+\Psi_{\mathrm{geom}}\ln\left(\varepsilon_{W}^{-}\right)\right]$
General sigmoidal (Sg) mean of upper and lower Wiener bounds [4]	$\varepsilon_{\mathrm{eff}}=\left(1-\delta_{0,N}\right){\left[\left(1-P\right){\left(\varepsilon_{W}^{+}\right)}^{N}+P{\left(\varepsilon_{W}^{-}\right)}^{N}\right]}^{\frac{1}{N}}$ $+\delta_{0,N}\left\{\exp\left[\left(1-P\right)\ln\left(\varepsilon_{W}^{+}\right)+P\ln\left(\varepsilon_{W}^{-}\right)\right]\right\}$ $\mathrm{where}\;\delta_{0,N}=\{\begin{array}{c} 1\\ 0 \end{array}\begin{array}{c} \mathrm{if}\;N=0\\ \mathrm{otherwise} \end{array}$

**Figure A2:**
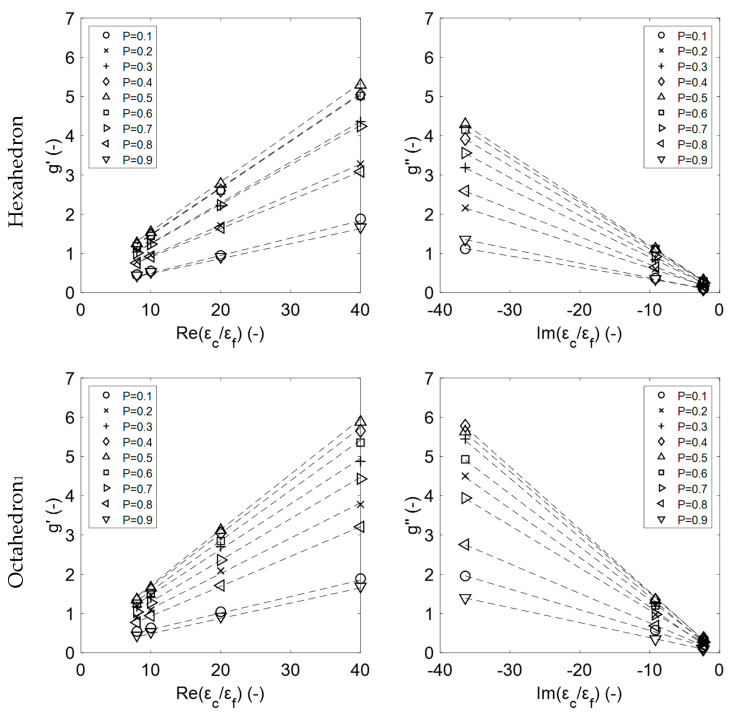

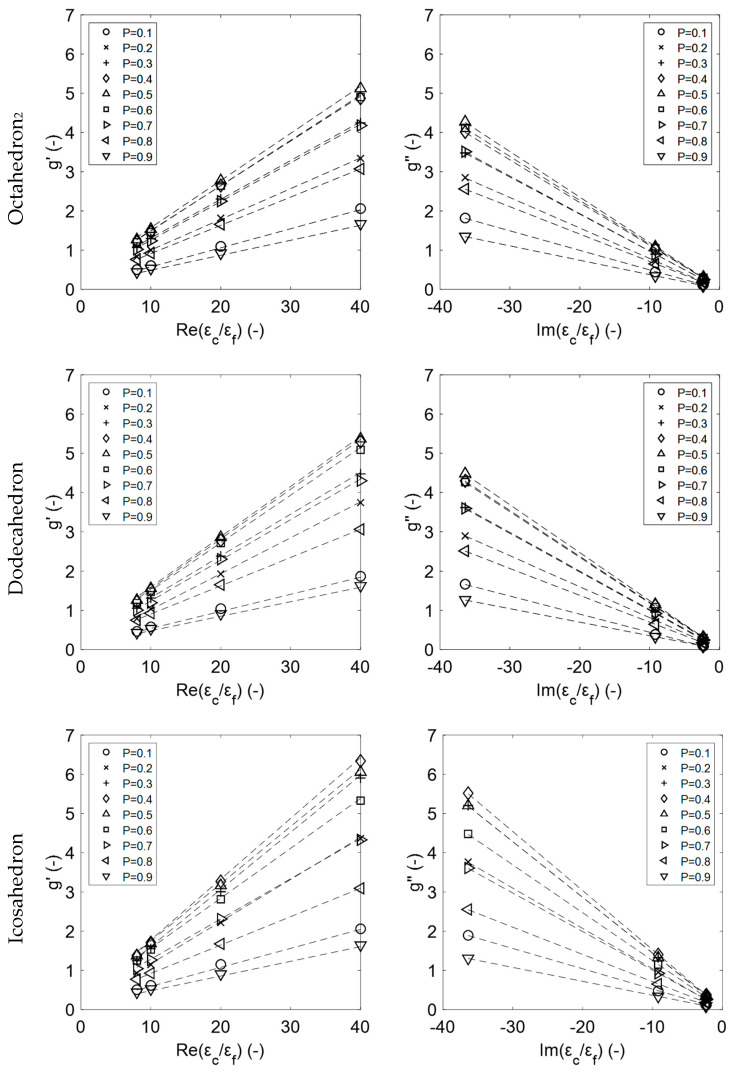
Complex-valued correlation parameter g of the Platonic foams vs. complex permittivity contrast ratio (all linear regressions are R^2^ > 0.99).

**Figure A3:**
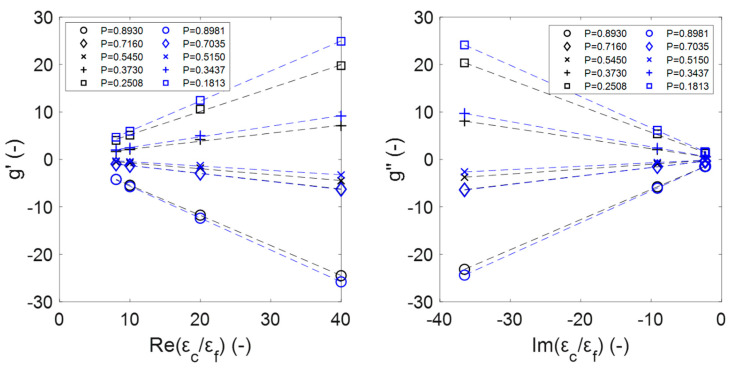
Effect of the permittivity contrast ratio on real (**left**) and imaginary (**right**) part of the complex-valued correlation parameter g of the sintered foams with 20 ppi (black symbols) and 30 ppi (blue symbols) pore density (all linear regressions are R^2^ > 0.99).

## Data Availability

Data is contained within the article.

## Appendix A

### Appendix A.1. Skeletal Volume Calculation Sequence for Platonic Foams

The following points summarize the calculation sequence for the volume of the skeletons of the studied Platonic foams (an example is presented below in Appendix A.2). The following parameters are known about the Platonic foam: the number of intersecting cylinders at each side $N_{\mathrm{cyl}}$, the interior angle of the face polygon $\theta_{P}$, the cylinder length $L_{\mathrm{cyl}}$, and the cylinder diameter $d_{\mathrm{cyl}}$.

- Calculate the volume of all cylinders at each intersection: $V_{A}=N_{\mathrm{cyl}}\pi d_{\mathrm{cyl}}{}^{2}L_{\mathrm{cyl}}/4$.
- Calculate the volumes of two intersecting cylinders at $\theta_{P}$: $V_{B}=2d_{\mathrm{cyl}}{}^{3}/\left(3\sin\theta_{P}\right)$.
- Calculate the sum of the volumes of $V_{B}$: $V_{C}=N_{\mathrm{cyl}}V_{B}$.
- Calculate the volume of all symmetrical intersections of the right circular cylinders at the vertex $V_{D}$. Refer to [26] or use cylindrical algebraic decomposition [27].
- Calculate the volume of the total solid by using the inclusion-exclusion principle: $V_{E}=V_{A}$ − $V_{C}$ + $V_{D}$.
- Calculate the percentage $N_{\mathrm{Per}}$ of $V_{E}$ enclosed in the PCA. Use symmetry (e.g., hexahedral or octahedral skeleton) or modeling software (e.g., computer-aided design).
- Calculate the total polyhedron volume inside the PCA: $V_{F}=N_{\mathrm{Per}}V_{E}$.
- Calculate the volume of all inserted spherical triangle segments: $V_{G}=n_{\mathrm{seg}}d_{\mathrm{cyl}}{}^{3}E/24$, where E is the spherical excess, which is solved by L’Huilier’s theorem [28], and $n_{\mathrm{seg}}$ is the number of segments added to the skeleton.
- Calculate the volume of the Platonic skeleton: $V_{P}=V_{F}+V_{G}$.

### Appendix A.2. Example of Skeletal Volume Calculation of Hexahedral Foams

The volume of the hexahedral foam ($N_{c}=3,\;\theta_{P}=90{}^{\circ}$) is obtained from:

- $V_{A}=3/4\pi d_{\mathrm{cyl}}{}^{2}L_{\mathrm{cyl}}$
- $V_{B}=2d_{\mathrm{cyl}}{}^{3}/3$
- $V_{C}=2d_{\mathrm{cyl}}{}^{3}$
- $V_{D}=\left(2-\sqrt{2}\right)d_{\mathrm{cyl}}{}^{3}$
- $V_{E}=3/4\pi d_{\mathrm{cyl}}{}^{2}L_{\mathrm{cyl}}-\sqrt{2}d_{\mathrm{cyl}}{}^{2}$
- $N_{R}=1$ (by symmetry, the whole solid can be arranged to give the PCA)
- $V_{F}=V_{E}$
- $V_{G}=0$ (for the hexahedral foam, a non-spherical pyramid segment is needed)
- $V_{P}=3/4\pi d_{\mathrm{cyl}}{}^{2}L_{\mathrm{cyl}}-\sqrt{2}d_{\mathrm{cyl}}{}^{2}$

The result from (IX) for $V_{P}$ is given in Table A1 for the hexahedral foam.

### Appendix A.3. Deriving the Permittivity Mixture-Relation for Platonic Foams

We propose a mixing relation for the $\varepsilon_{\mathrm{eff}}$ of Platonic foams that is based on the analysis of their permittivities. The amplitude of the polarizability α of a single scatterer dipole is:

(A1) $$\alpha =p/E_{e},$$

where p is the induced dipole moment of a single inclusion in a uniform local electric field with the magnitude $E_{e}$. In previous calculations [29,30], it was shown that for inclusions with minimal symmetry defects—which holds for Platonic solids—their α is a simple dyadic (a scalar for $\varepsilon \left(\varepsilon^{\prime \prime }=0\right)$), which is obtained by solving its electrostatic field. Note that to consider the dielectric loss ($\varepsilon^{\prime \prime }\neq 0$), the polarizability has to be represented as a complex number. Physically, this description may occur in an oscillating electric field (e.g., microwaves) with a damping velocity response (relaxation time [31]) of the induced dipole moment. This methodology is accurate but requires significant numerical effort for complex geometries, such as the Platonic foams. Thus, it was better to start from the macroscopic domain (assuming that the loss mechanism was the same in bulk and foam materials), in search of a complex-valued correlation parameter g that contained the topological details of the micro-geometry in terms of an expression of the volume fraction of the skeleton (1−P) and fluid (P), as well as the effective and bulk permittivities. Observe that at P<0.5 the fluid corresponds to the inclusion medium and the skeleton to the guest medium, and vice versa at P≥0.5. Accordingly, the parameter g for ceramic and fluid properties was proposed as:

(A2) $$g=\left[\begin{array}{cc} \alpha_{c} & \alpha_{f} \end{array}\right]\cdot \left[\begin{array}{c} \beta_{c}\\ \beta_{f} \end{array}\right],$$

where $\alpha_{c},\alpha_{f}$ and $\beta_{c},\beta_{f}$ are variable expressions in terms of the permittivity and volume fraction of the ceramic and fluid phase, respectively. The following expressions were proposed to assess $\alpha_{c},\alpha_{f}$, $\beta_{c},\beta_{f}$ as:

(A3) $$\alpha_{c}=\frac{q_{0}\varepsilon_{\mathrm{eff}}+q_{2}\varepsilon_{c}}{q_{4}\varepsilon_{c}+q_{5}\varepsilon_{f}},$$

(A4) $$\beta_{c}=\frac{\sum_{u=n_{1}}^{m_{1}}q_{6}{\left(1-P\right)}^{u}}{\sum_{k=n_{3}}^{m_{3}}q_{8}{\left(1-P\right)}^{k}+\sum_{j=n_{4}}^{m_{4}}q_{9}P^{j}},$$

(A5) $$\alpha_{f}=\frac{q_{1}\varepsilon_{\mathrm{eff}}+q_{3}\varepsilon_{f}}{q_{4}\varepsilon_{c}+q_{5}\varepsilon_{f}},$$

(A6) $$\beta_{f}=\frac{\sum_{j=n_{2}}^{m_{2}}q_{7}P^{j}}{\sum_{k=n_{5}}^{m_{3}}q_{8}{\left(1-P\right)}^{k}+\sum_{j=n_{4}}^{m_{4}}q_{9}P^{j}},$$

where all q-parameters are real-valued integers. At the porosity limits, P=0 and P=1, the material corresponds completely to the ceramic and the fluid, which have no microstructure (g=0). Then, the numerator of the assessed expressions were selected to comply with the natural bounds $\varepsilon_{\mathrm{eff}}\left(P=0\right)=\varepsilon_{c}$ and $\varepsilon_{\mathrm{eff}}\left(P=1\right)=\varepsilon_{f}$ by setting $q_{0}=-q_{2}$, $q_{1}=-q_{3}$, $n_{1}\neq 0$ and $n_{2}\neq 0$, while for the denominators, the expressions are flexible enough to be evaluated with any combination of bulk permittivities and volume fractions. For each set of parameters, several relation equations were deduced, in which g is given as a complex expression defined as:

(A7) $$g\left(P,\varepsilon_{c},\varepsilon_{f}\right)=g^{\prime }-jg^{\prime \prime }.$$

For isotropic materials g must be proportionally dependent to the permittivities of the mixture. On this basis, a selection condition was defined for the mixing relation to give a linear dependence of g to the permittivity contrast as:

(A8) g′=Re(εcεf)gm′+g0′,

(A9) g″=−Im(εcεf)gm″−g0″,

where $g_{0}^{\prime }$, $g_{0}^{\prime \prime }$ are the g values when $\varepsilon_{c}/\varepsilon_{f}\to 0$ and $g_{m}^{\prime }$ and $g_{m}^{\prime \prime }$ correspond to the values of the slopes. Next, an evaluation routine was setup using MATLAB (R2020a, Mathworks, USA). After some iterations using the effective permittivities that were obtained from the simulations and limiting k≤2 and j≤2 (due to time and computer resources), an expression was obtained, which satisfies the selection condition of linearity ($R^{2}>0.99$, see Figure A2 of the Appendix A) and is given as:

(A10) $$g=\left[\begin{array}{cc} \frac{\left(\varepsilon_{\mathrm{eff}}-\varepsilon_{c}\right)}{\varepsilon_{f}} & \frac{\left(\varepsilon_{\mathrm{eff}}-\varepsilon_{f}\right)}{\varepsilon_{f}} \end{array}\right]\cdot \left[\begin{array}{c} \frac{{\left(1-P\right)}^{2}}{\left({\left(1-P\right)}^{2}+2P\right)}\\ \frac{2P}{\left({\left(1-P\right)}^{2}+2P\right)} \end{array}\right].$$

The equation can be rewritten as:

(A11) $$g=\frac{1}{\varepsilon_{f}}\left[\frac{2P}{\left(1+P^{2}\right)}\left(\varepsilon_{c}-\varepsilon_{f}\right)-\left(\varepsilon_{c}-\varepsilon_{\mathrm{eff}}\right)\right]\;\mathrm{or}$$

(A12) $$\varepsilon_{\mathrm{eff}}=\frac{-2P}{\left(1+P^{2}\right)}\left(\varepsilon_{c}-\varepsilon_{f}\right)+\left(\varepsilon_{c}+g\varepsilon_{f}\right).$$

From Figure A2 of the Appendix A, it is observed that none of the Platonic foams has a symmetrical inclusion–background morphology, i.e., Re(g(P))≠Re(g(1−P)). Most symmetry is obtained for the hexahedron skeleton (see Figure A2 of the Appendix A), where the morphology of the skeleton only differs due to the cylindrical struts. Please note that a full symmetry would be obtained by using rectangular struts.

Unlike spheres and ellipsoids, which are common structures of the EMA, the micro-structure of Platonic foams changes with porosity as a result of the interference of the geometry of the skeleton elements (e.g., joints). This gives $g_{0}^{\prime }$, $g_{0}^{\prime \prime }$, $g_{m}^{\prime }$ and $g_{m}^{\prime \prime }$ an exact value at a certain porosity. However, to consider the whole porosity range P∈[0,1], these parameters are expressed as:

(A13) $$Y=\overset{6}{\underset{k=1}{\sum }}a_{k}P^{k},$$

where Y = $\left\{g_{0}^{\prime },\;g_{0}^{\prime \prime },\;g_{m}^{\prime },g_{m}^{\prime \prime }\right\}$ and their respective coefficients $a_{k}$ are summarized in Table A2 of the Appendix A. Note that the proposed relation can only be applied for the valid porosity range (which preserves the Platonic structure, as shown in Table 2). The extension of this approach to derive a relation for sintered foams is illustrated in Appendix A.4 of the Appendix A.

### Appendix A.4. Extending the Proposed Permittivity Mixture Relation for Sintered Foams

Although the new relation was obtained directly from the analysis of the Platonic foam data, it also presents a linear relation between g and $\varepsilon_{c}/\varepsilon_{f}$ for sintered foams (see Figure A3 of the Appendix A). However, in contrast to the Platonic foams, the real and imaginary parts of $g\left(P,\varepsilon_{c},\varepsilon_{f}\right)$ (Equation (A7) of the Appendix A) at different pore densities (20 and 30 ppi) are well approximated by a simplified expression as:

(A14) $$g\left(P,\varepsilon_{c},\varepsilon_{f}\right)\;=\left(\varepsilon_{c}/\varepsilon_{f}\right)P{\left(1-P\right)}^{3/2}.$$

Then, substituting Equation (A14) into Equation (A12) gives:

(A15) $$\varepsilon_{\mathrm{eff}}=\frac{-2P}{\left(1+P^{2}\right)}\left(\varepsilon_{c}-\varepsilon_{f}\right)+\varepsilon_{c}\left(1+P{\left(1-P\right)}^{3/2}\right).$$

We refer to Equation (A15) as the open-cell foam (OCF) relation since it estimates the effective permittivity of the open-cell foams.

### Appendix A.5. Tables and Figures

**Table A1:** Formulas to calculate the volume of the Platonic skeletons.

Platonic Skeleton	$\mathrm{Relation}\;\mathrm{between}\;L_{cyl}$ $\;\mathrm{and}\;L_{PCA}.$	Volume of the Platonic skeleton
Hexahedron	$L_{\mathrm{cyl}}=L_{\mathrm{PCA}}$	$V_{p}=\frac{3}{4}\pi d_{\mathrm{cyl}}{}^{2}L_{\mathrm{cyl}}-\sqrt{2}d_{\mathrm{cyl}}{}^{3}$
Octahedron_1_	$L_{\mathrm{cyl}}=L_{\mathrm{PCA}}/\sqrt{2}$	$V_{p}=3\pi d_{\mathrm{cyl}}{}^{2}L_{\mathrm{cyl}}-\left(\frac{59}{8}+\frac{93}{100}\left(\sqrt{8}-\sqrt{6}\right)\right)d_{\mathrm{cyl}}{}^{3}$
Octahedron_2_	$L_{\mathrm{cyl}}=L_{\mathrm{PCA}}/\sqrt{2}$	$V_{p}=6\pi d_{\mathrm{cyl}}{}^{2}L_{\mathrm{cyl}}-\left(\frac{2939}{155}+\frac{93}{100}\left(\sqrt{8}-\sqrt{6}\right)\right)d_{\mathrm{cyl}}{}^{3}$
Dodecahedron	$L_{\mathrm{cyl}}=\frac{L_{\mathrm{PCA}}}{\left(3+\sqrt{5}\right)}$	$V_{p}=\frac{27}{2}\pi d_{\mathrm{cyl}}{}^{2}L_{\mathrm{cyl}}-\left(\frac{152}{7\sin\left(108\right)}-10\tan\left(54\right)\right)d_{\mathrm{cyl}}{}^{3}$
Icosahedron	$L_{\mathrm{cyl}}=\frac{L_{\mathrm{PCA}}}{2\left(1+\sqrt{5}\right)}$	$V_{p}=27\pi d_{\mathrm{cyl}}{}^{2}L_{\mathrm{cyl}}-\left(\frac{1}{3}\pi +\frac{15}{2}\sin\left(\frac{151}{3}\right)-\frac{95}{\sqrt{12}}\right)d_{\mathrm{cyl}}{}^{3}$

**Table A2:** Parameters used to calculate $g_{0}^{\prime }$, $g_{0}^{\prime \prime }$, $g_{m}^{\prime }$ and $g_{0}^{\prime \prime }$.

Variable	$a_{1}$	$a_{2}$	$a_{3}$	$a_{4}$	$a_{5}$	$a_{6}$
Hexahedron						
$g\prime_{m}$	0.5229	−0.9951	2.4460	−4.7673	3.9566	−1.1630
$g_{0}^{\prime }$	0.2775	4.7271	−14.1449	15.0204	−6.6742	0.7939
$g_{m}^{\prime \prime }$	0.2889	−0.0356	0.8263	−3.3594	3.3200	−1.0402
$g_{0}^{\prime \prime }$	−1.2278	10.1408	−36.4260	63.3016	−52.0799	16.2911
Octahedron_1_						
$g_{m}^{\prime }$	0.4008	0.6699	−3.2970	3.9169	−2.2435	0.5528
$g_{0}^{\prime }$	2.1613	−7.0162	17.1386	−28.2326	23.5875	−7.6392
$g_{m}^{\prime \prime }$	0.3209	4.0884	−19.4308	33.9140	−27.2604	8.3683
$g_{0}^{\prime \prime }$	−2.0185	26.2707	−105.0485	185.5296	−151.8010	47.0699
Octahedron_2_						
$g_{m}^{\prime }$	0.6546	−2.1904	6.0160	−9.7020	7.2485	−2.0266
$g_{0}^{\prime }$	0.3698	7.6377	−26.3063	34.1470	−20.6464	4.7987
$g_{m}^{\prime \prime }$	0.7183	−2.8553	8.1037	−12.6555	9.2101	−2.5214
$g_{0}^{\prime \prime }$	0.1978	−2.4611	7.5172	−10.0237	6.2255	−1.4557
Dodecahedron						
$g_{m}^{\prime }$	0.4617	0.3546	−3.9082	7.6496	−7.0236	2.4661
$g_{0}^{\prime }$	1.4799	−10.1720	50.1347	−109.2526	103.7717	−35.9641
$g_{m}^{\prime \prime }$	0.6052	−1.7991	4.9208	−8.1320	5.9922	−1.5869
$g_{0}^{\prime \prime }$	0.1169	−3.0237	12.1921	−20.3098	15.4249	−4.4004
Icosahedron						
$g_{m}^{\prime }$	0.2213	4.1649	−17.8917	29.0420	−21.8497	6.3132
$g_{0}^{\prime }$	2.4320	−21.2239	84.7916	−152.1911	124.4845	−38.2943
$g_{m}^{\prime \prime }$	0.3270	2.8913	−12.9887	20.3933	−14.6653	4.0423
$g_{0}^{\prime \prime }$	−0.1270	0.6904	−2.6267	4.5291	−3.4173	0.9512

## References

[1] Wan T., Liu Y., Zhou C., Chen X., Li Y. (2020). Fabrication, properties, and applications of open-cell aluminum foams: A review. J. Mater. Sci. Technol..

[2] Zalucky J., Schubert M., Lange R., Hampel U. (2017). Dynamic Liquid–Solid Mass Transfer in Solid Foam Packed Reactors at Trickle and Pulse Flow. Ind. Eng. Chem. Res..

[3] Bracconi M., Ambrosetti M., Maestri M., Groppi G., Tronconi E. (2018). A fundamental investigation of gas/solid mass transfer in open-cell foams using a combined experimental and CFD approach. Chem. Eng. J..

[4] Pabst W., Hříbalová S. (2019). Describing the Effective Conductivity of Two-Phase and Multiphase Materials via Weighted Means of Bounds and General Power Means. JOM.

[5] Torquato S., Haslach H. (2002). Random Heterogeneous Materials: Microstructure and Macroscopic Properties. Appl. Mech. Rev..

[6] Diani A., Bodla K.K., Rossetto L., Garimella S.V. (2014). Numerical Analysis of Air Flow through Metal Foams. Energy Procedia.

[7] Pickles A.J., Steer M.B. (2013). Effective Permittivity of 3-D Periodic Composites With Regular and Irregular Inclusions. IEEE Access.

[8] Pabst W., Gregorová E., Uhlířová T. (2015). Microstructure characterization via stereological relations—A shortcut for beginners. Mater. Charact..

[9] Lorensen W.E., Cline H.E. (1987). Marching cubes: A high resolution 3D surface construction algorithm. Proc. Siggraph.

[10] Hernandez J.N.C., Link G., Soldatov S., Füssel A., Schubert M., Hampel U. (2020). Experimental and numerical analysis of the complex permittivity of open-cell ceramic foams. Ceram. Int..

[11] Rueden C.T., Schindelin J., Hiner M.C., Dezonia B.E., Walter A.E., Arena E.T., Eliceiri K.W. (2017). ImageJ2: ImageJ for the next generation of scientific image data. BMC Bioinform..

[12] Ollion J., Cochennec J., Loll F., Escudé C., Boudier T. (2013). TANGO: A generic tool for high-throughput 3D image analysis for studying nuclear organization. Bioinformatics.

[13] Dougherty R., Kunzelmann K.-H. (2007). Computing Local Thickness of 3D Structures with ImageJ. Microsc. Microanal..

[14] Atiyah M., Sutcliffe P. (2003). Polyhedra in Physics, Chemistry and Geometry. Milan J. Math..

[15] Numan A.B., Sharawi M.S. (2013). Extraction of Material Parameters for Metamaterials Using a Full-Wave Simulator [Education Column]. IEEE Antennas Propag. Mag..

[16] Hernandez J.N.C., Lecrivain G., Schubert M., Hampel U. (2019). Droplet Retention Time and Pressure Drop in SiSiC Open-Cell Foams Used as Droplet Separation Devices: A Numerical Approach. Ind. Eng. Chem. Res..

[17] Mishchenko M.I., Dlugach J.M., Liu L. (2016). Applicability of the effective-medium approximation to heterogeneous aerosol particles. J. Quant. Spectrosc. Radiat. Transf..

[18] Baker-Jarvis J., Janezic M., Riddle B., Johnk R., Holloway C., Geyer R., Grosvenor C. (2005). Measuring the Permittivity and Permeability of Lossy Materials: Solids, Liquids, Metals, and negative-Index Materials.

[19] (2020). MATLAB 2020.

[20] MATLAB 2021, Fit a Model to Complex-Valued Data. https://www.mathworks.com/help/optim/ug/fit-model-to-complex-data.html.

[21] Camerucci M.A., Urretavizcaya G., Castro M., Cavalieri A. (2001). Electrical properties and thermal expansion of cordierite and cordierite-mullite materials. J. Eur. Ceram. Soc..

[22] Westphal W., Sils A. (1972). Dielectric Constant and Loss Data.

[23] Ratanadecho P., Aoki K., Akahori M. (2002). The characteristics of microwave melting of frozen packed beds using a rectangular waveguide. IEEE Trans. Microw. Theory Tech..

[24] He X., Zhou J., Jin L., Long X., Wu H., Xu L., Gong Y., Zhou W. (2020). Improved Dielectric Properties of Thermoplastic Polyurethane Elastomer Filled with Core–Shell Structured PDA@TiC Particles. Materials.

[25] McKeen L.W. (2012). Chapter 1—Introduction to Plastics and Polymers. Plastics Design Library, Film Properties of Plastics and Elastomers.

[26] Moore M. (1974). Symmetrical intersections of right circular cylinders. Math. Gaz..

[27] Strzebonski Cylindrical Algebraic Decomposition. From MathWorld—A Wolfram Web Resour. Creat. by Eric W. Weisstein. (n.d.). https://mathworld.wolfram.com/CylindricalAlgebraicDecomposition.html.

[28] Weisstein E.W. L’Huilier’s Theorem. From MathWorld—A Wolfram Web Resour. (n.d.). https://mathworld.wolfram.com/LHuiliersTheorem.html.

[29] Sihvola A., Yla-Oijala P., Jarvenpaa S., Avelin J. (2004). Polarizabilities of Platonic Solids. IEEE Trans. Antennas Propag..

[30] Sihvola A. (2007). Dielectric Polarization and Particle Shape Effects. J. Nanomater..

[31] Martínez E.S., Calleja R.D., Gunßer W. (1992). Complex polarizability as used to analyze dielectric relaxation measurements. Colloid Polym. Sci..

